# Transcriptome Comparison of Human Neurons Generated Using Induced Pluripotent Stem Cells Derived from Dental Pulp and Skin Fibroblasts

**DOI:** 10.1371/journal.pone.0075682

**Published:** 2013-10-03

**Authors:** Jian Chen, Mingyan Lin, John J. Foxe, Erika Pedrosa, Anastasia Hrabovsky, Reed Carroll, Deyou Zheng, Herbert M. Lachman

**Affiliations:** 1 Department of Psychiatry and Behavioral Sciences, Albert Einstein College of Medicine, Bronx, New York, United States of America; 2 Department of Genetics, Albert Einstein College of Medicine, Bronx, New York, United States of America; 3 Dominick Purpura Department of Neuroscience, Albert Einstein College of Medicine, Bronx, New York, United States of America; 4 Department of Pediatrics, Albert Einstein College of Medicine, Bronx, New York, United States of America; 5 Department of Neurology, Albert Einstein College of Medicine, Bronx, New York, United States of America; 6 Department of Medicine, Albert Einstein College of Medicine, Bronx, New York, United States of America; Instituto Butantan, Brazil

## Abstract

Induced pluripotent stem cell (iPSC) technology is providing an opportunity to study neuropsychiatric disorders through the capacity to grow patient-specific neurons *in vitro*. Skin fibroblasts obtained by biopsy have been the most reliable source of cells for reprogramming. However, using other somatic cells obtained by less invasive means would be ideal, especially in children with autism spectrum disorders (ASD) and other neurodevelopmental conditions. In addition to fibroblasts, iPSCs have been developed from cord blood, lymphocytes, hair keratinocytes, and dental pulp from deciduous teeth. Of these, dental pulp would be a good source for neurodevelopmental disorders in children because obtaining material is non-invasive. We investigated its suitability for disease modeling by carrying out gene expression profiling, using RNA-seq, on differentiated neurons derived from iPSCs made from dental pulp extracted from deciduous teeth (T-iPSCs) and fibroblasts (F-iPSCs). This is the first RNA-seq analysis comparing gene expression profiles in neurons derived from iPSCs made from different somatic cells. For the most part, gene expression profiles were quite similar with only 329 genes showing differential expression at a nominally significant p-value (p<0.05), of which 63 remained significant after correcting for genome-wide analysis (FDR <0.05). The most striking difference was the lower level of expression detected for numerous members of the all four *HOX* gene families in neurons derived from T-iPSCs. In addition, an increased level of expression was seen for several transcription factors expressed in the developing forebrain (*FOXP2, OTX1, and LHX2*, for example). Overall, pathway analysis revealed that differentially expressed genes that showed higher levels of expression in neurons derived from T-iPSCs were enriched for genes implicated in schizophrenia (SZ). The findings suggest that neurons derived from T-iPSCs are suitable for disease-modeling neuropsychiatric disorder and may have some advantages over those derived from F-iPSCs.

## Introduction

We and other groups are using induced pluripotent stem cells (iPSCs) for *in vitro* disease modeling in a variety of neuropsychiatric disorders, including schizophrenia (SZ) and autism spectrum disorders (ASD) [1]–[10]. In addition to their utility for disease modeling in terms of identifying patient vs control differences in gene expression, morphology, synaptic architecture, and neuronal function, iPSCs can also be used to study human neurogenesis *in vitro*, which is particularly relevant to SZ and ASD considering that both have a neurodevelopmental basis. A variety of cell types have been used for iPSC reprogramming, but fibroblasts obtained from skin biopsy samples have been the mainstay for neuropsychiatric disorders so far. This presents a potential obstacle for modeling genetically-based childhood disorders. Although iPSCs have been developed from children with ASD and other developmental problems using fibroblasts and more recently, peripheral blood, it is somewhat problematic because such children often fear medical procedures, even routine phlebotomy [3]–[5], [8]. Thus, alternative sources of cells for iPSC reprogramming obtained by non-invasive means would be useful. Also, because iPSCs may retain some cell-of-origin epigenetic marks, testing differentiating neurons derived from iPSCs generated from various somatic cells to assess their utility for modeling neuropsychiatric disorders is important. One potential source of somatic cells is dental pulp derived from deciduous teeth. iPSCs derived from dental pulp (T-iPSCs) display typical molecular and cellular features of pluripotency, and have been shown to differentiate into neurons [11]–[13]. However, a more detailed molecular profile is needed to assess the similarity of iPSC-derived neurons from different somatic tissues/cells and their potential use for modeling neuropsychiatric disorders.

Consequently, we have carried out an extensive gene expression profiling analysis of neurons derived from T-iPSCs using whole transcriptome profiling (RNA-Seq) and compared that with neurons derived iPSCs made from fibroblasts (F-iPSCs). Our gene expression profiling studies show a high degree of correlation for the two sources of neurons. However, there are subtle differences that might influence the decision to use T-iPSCs or F-iPSCs for some neuropsychiatric disorders.

## Materials and Methods

### Development of iPSCs from Dental Pulp and Skin Fibroblasts

The study was approved by the Albert Einstein College of Medicine Institutional Review Board. Written informed consent was obtained for subjects undergoing a skin biopsy, which was carried out by a board-certified dermatologist. For the tooth sample, signed written assent was provided by the subject, who was 12 years old at the time; the assent was countersigned by a parent. The tooth sample was lost naturally and not extracted. Consent for the skin biopsy samples was obtained by a senior member of the research team (the corresponding author). Assent for the tooth sample was obtained by a senior level associate (Ph.D. level) of one of the co-authors (JJF). T-iPSC lines (TIPS4 and TIPS4-C5) were generated from dental pulp cells harvested from a molar tooth that was naturally shed by the subject, a 12 year old healthy Caucasian male. The method for collecting deciduous teeth, extracting dental pulp, and growing these cells in culture is described in greater detail in Supplemental Methods (Text S1). Fibroblasts were obtained from skin biopsies performed in consenting adults by a board-certified dermatologist. The detailed procedure for growing fibroblasts in preparation for reprogramming into iPSCs is also in the Text S1. The F-iPSC lines referred to throughout the paper as F-iPSC1 and F-iPSC2 were derived from a 30 year old female and a 58 year old male, respectively. The development of these lines was previously described [1], [2], [14].

iPSC reprogramming was carried out by nucleofection. Briefly, one vial of cells was thawed out and placed in a T75 flask in “tooth medium” (see Text S1 for formula) and fed every 2 days. Cells were grown to ∼50% confluence (∼4–5 days), after which they were trypsinized and subjected to nucleofection (∼6 x10^5^ cells). Reprogramming was carried out using an Amaxa 4D-Nucleofector (P2 Primary Cell Kit from Lonza cat# V4XP-2012, Program FF-135) with non-integrating plasmids containing *OCT4, SOX2, KLF4, L-MYC, LIN28,* and a p53 shRNA vector (Addgene Cat. # 27077, 27078, 27080), according to Okita et al., with some modifications [1], [2], [14], [15]. iPSCs were grown on Matrigel plates and maintained in mTeSR1 medium (Stem Cell Technologies).

### Germ Line Markers and Establishing Pluripotency by *in vitro* Differentiation

To assess pluripotency, iPSCs were stained with Ab against Tra-1-60, Tra-1-81, SSEA3 and SSEA4, which are expressed in pluripotent stem cells. In addition, for TIPS4, the capacity to differentiate into all 3 germ layers was established by *in vitro* assays, as previously described [1], [2], [14]. The markers desmin (mesoderm), α-fetoprotein (endoderm), and βIII-tubulin (ectoderm) were used [16]–[18] (Figure S1). For TIPS4-C5, pluripotency was established in a similar manner (Figure S2); germ line differentiation was confirmed by the capacity to form embryoid bodies, differentiate into functional neurons (described below), and the expression of germ-line markers by PCR following differentiation (*AFP;* endoderm, *ACTA2;* mesoderm, and *MAP2;* ectoderm (not shown). Immunocytochemistry was carried as previously described [19], [20]. A list of the antibodies used in the study is shown in Text S1.

### Neuronal Differentiation

Neurons were derived from neural progenitor cells (NPCs) as described by Marchetto et al. with slight modifications [1], [9]. A detailed description of the protocol is in Text S1.

### RNA-Seq

RNA-seq was carried out on iPSCs, NPCs and day 14 neurons derived from TIPS4 and TIPS4-C5, and day 14 neurons from F-iPSC1 and F-iPSC2. Total RNA was isolated from cells using the miRNeasy Kit (Qiagen) according to the manufacturer’s protocol. An additional DNAse1 digestion step was performed to ensure that the samples were not contaminated with genomic DNA. RNA purity was assessed using the Agilant 2100 Bioanalyzer (Beijing Genomics Institute). Each RNA sample had an A260:A280 ratio above 1.8, a RIN>9, and an A260:A230 ratio above 2.2. Briefly, total RNA was converted to cDNA using oligo dT, which was then used for Illumina sequencing library preparation. Paired end RNA-seq was carried on an Illumina HiSeq 2000. We obtained 90-bp mate-paired reads from DNA fragments of with an average size of 250-bp (standard deviation for the distribution of inner distances between mate pairs is approximately 50 bp). RNA-Seq reads were aligned to the human genome (GRCh37/hg19) using the software TopHat (version 2.0.8) [21]. We counted the number of fragments mapped to each gene annotated in the GENCODE database (version 15) [22]. The category of transcripts is described at http://vega.sanger.ac.uk/info/about/gene_and_transcript_types.html. Transcript abundances were measured in FPKM (fragments per kilobase of exon per million fragments mapped). We used DESeq (an R package developed by Anders and Huber) to evaluate differential expression from count data [23]. We used DESeq (an R package developed by Anders and Huber) to evaluate differential expression from count data {{2762 Anders,S. 2010}}. Specifically, DESeq models the variance in fragment counts across replicates using the negative binomial distribution and tests whether, for a given gene, the change in expression strength between the two experimental conditions is significantly large as compared to the variation within each replicate group. In the end, only genes with average FPKMs larger than 1 across samples were considered for differential expression. The number of reads obtained from the RNA-seq runs for each sample and the fraction that could be aligned to the human genome was consistent across samples (Table S1). In addition, the correlation coefficients were very high for biological replicates (Table S1). Sequence data can be accessed at the NCBI’s (National Center for Biotechnology Information) Gene Expression Omnibus (accession number GSE43143).

### Reverse Transcribed PCR (RT-PCR) and Quantitative Real-time PCR (qPCR)

Reverse transcribed PCR (RT-PCR) was performed using a OneStep RT-PCR Kit (Qiagen, Valencia, CA) according to the manufacturer’s instructions. The cDNA was generated using an iScriptTM cDNA synthesis Kit (Bio-RAD, Hercules, CA) and subsequently used as a template for quantitative qPCR, which was carried out with an ABI 7900HT Real-Time PCR System instrument (Applied Biosystems, Foster City, CA). Each reaction consisted of cDNA, primers, and SYBR Green PCR Master Mix (Applied Biosystems, Foster City, CA) in an 8 µl volume (primers used in this study are shown in Text S1). Relative changes in gene expression were calculated using the 2^−ΔΔCt^ method with β2-microglobulin (β2M) as a reference gene. The primers used in this study, as well as technical details are in Text S1.

### Electrophysiology

Whole cell recordings were made using a Multiclamp 700B (Molecular Devices, Sunnyvale, CA) in 35 day old differentiated cultures. Neuronal-like cells characterized by extended processes were chosen for recording. Low-resistance pipettes (3–5 MOhm) contained: 135 mM K gluconate, 6 mM NaCl, 10 mM HEPES, 1 mM EGTA, 0.5 mM CaCl2, 10 mM Glucose, 2 mM MgATP, 0.3 mM NaGTP pH 7.2. Cells were perfused with an extracellular solution containing 120 mM NaCl, 26 mM NaHCO_3_, 2.5 mM KCl, 1 mM NaH_2_PO_4_, 20 mM Glucose, 2.5 mM CaCl_2_, 1.3 mM MgSO_4_ and adjusted to pH 7.4 and infused with 95% O_2_/5% CO_2_. Data was acquired using Igor Pro Software (Wavemetrics, Lake Oswego, OR). The stability of series and input resistances were confirmed throughout the experiment. Signals were filtered at 2 KHz and digitized at 5 KHz. To analyze action potential generation, cells were held in current clamp at −75 mV and a 10–500 ms current of 100–200pA was injected to depolarize cells to threshold.

## Results

### Differentiation into Functional Neurons

**Figure 1:**
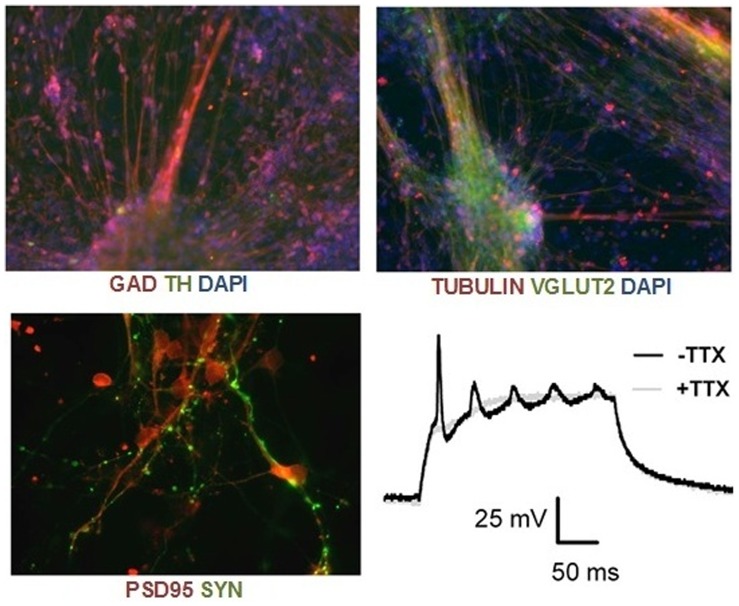
immunostaining for GAD, TH, βIII-tubulin and VGLUT2 in TIPS4 neurons (day 14), and PSD95 and synaptophysin (SYN) in day 56 neurons. Lower right: Train of action potentials with and without tetrodoxin (TTX) in day 35 neurons.

Dental pulp cells were cultured and reprogrammed into iPSCs, then induced to differentiate into neurons, as described in the methods section. In previous experiments using neurons derived from F-iPSCs, the differentiation protocol resulted in the production of a heterogeneous mix of glutamatergic and GABAergic neurons that express forebrain, midbrain and hindbrain transcription factors (TFs) [1]–[9]. A small fraction of cells (∼1%) express the dopamine marker TH (unpublished observations). TIPS4 produced a similar mix of neurons (Figure 1A, 1B). As the cells matured, robust staining for the pre and post synaptic glutamatergic markers synaptophsin and PSD95 was seen (Figure 1C). In addition, after exposure to a depolarizing current, a train of action potentials could be detected; sodium channel activation is responsible for the response, since it’s blocked by tetrodotoxin (TTX) (Figure 1D). These findings confirm that functional neurons can be developed from T-iPSCs.

### RNA-Seq

Whole genome transcriptome analysis (RNA-Seq) was carried out on TIPS4 and TIPS4-C5 iPSCs, NPCs and 14 day neurons following neuronal differentiation. The expression levels for 1368 genes were differentially expressed during the transition from iPSCs to NPCs (627 higher in NPCs; 741 lower in NPCs, FDR <0.05). A comparison between iPSCs and neurons showed 2543 differentially expressed genes (1286 increased in neurons; 1257 lower in neurons, FDR <0.05) (entire list of differentially expressed genes during differentiation at all 3 transition points [iPSCs to NPCs; iPSCs to neurons; NPCs to neurons can be found in Table S2, Table S3, Table -S4). Among the genes that showed substantial decreases in expression in NPCs and neurons were *POU5F1* (*OCT4*), LIN28A, *TDGF1*, which are expressed at high levels in embryonic and pluripotent stem cells, and are inactivated during differentiation (Table S2 and Table S3) [14], [24].

Also among the most down-regulated genes during the transition from iPSCs to NPCs were several long non-coding RNAs (lncRNAs) including ESRG (embryonic stem cell related gene), RP11-1144P22.1, LINC00678, and RP11-256I9.2 (Table 1). These could represent novel non-coding genes that help maintain pluripotency. Interestingly, LINC00678 is transcribed antisense to BDNF-AS, a lncRNA involved in the regulation of BDNF, which has been implicated in SZ, bipolar disorder (BD), major depression and Rett Syndrome [25]–[30].

**Table 1:** Top differentially expressed genes (fold change; FC) that decrease during differentiation into NPCs.

Gene	FPKM iPSCs	FPKM NPCs	FPKM iPSCs	FPKM NPCs	log2 FC	p-value	FDR
	TIPS4	TIPS4	TIPS4-C5	TIPS4-C5	NPCs/iPSCs		
RP11-256I9.2	2100.54	0.00	126.91	0.00	−17.77	9.31E-39	1.49E-35
RP11-132A1.3	42.89	0.00	39.36	0.00	−13.01	1.42E-24	6.59E-22
ESRG	439.88	0.08	374.93	0.03	−12.82	2.71E-56	9.76E-53
RP11-1144P22.1	192.17	0.05	126.90	0.00	−12.43	1.51E-38	1.97E-35
RP11-277P12.10	39.44	0.00	13.94	0.00	−12.38	1.71E-08	9.11E-07
PRDM14	22.11	0.00	19.11	0.00	−12.01	3.22E-31	2.32E-28
CTD-2142D14.1	21.65	0.00	15.39	0.00	−11.86	1.60E-11	1.57E-09
TRIML2	13.32	0.00	19.97	0.00	−11.70	6.21E-11	5.54E-09
FOXH1	81.94	0.03	34.75	0.00	−11.70	1.91E-31	1.44E-28
CTD-2501M5.1	20.49	0.00	11.22	0.00	−11.63	1.02E-13	1.47E-11
LINC00678	184.49	0.05	111.91	0.04	−11.60	3.09E-37	3.71E-34
TDGF1	541.72	0.31	422.70	0.03	−11.43	4.40E-50	1.27E-46
VRTN	41.60	0.01	29.62	0.01	−11.33	1.65E-39	2.97E-36
RP1-46F2.2	11.17	0.00	9.79	0.00	−11.03	2.64E-04	4.55E-03
DPEP3	9.15	0.00	8.92	0.00	−10.82	2.46E-17	5.62E-15
ZFP42	87.65	0.07	56.90	0.00	−10.81	1.32E-38	1.90E-35
RP11-469A15.2	10.63	0.00	7.11	0.00	−10.79	6.98E-06	2.00E-04
CTD-2306M5.1	8.55	0.00	7.92	0.00	−10.69	3.14E-06	9.98E-05
DAZL	8.51	0.00	7.19	0.00	−10.62	2.17E-12	2.58E-10
CCL26	6.35	0.00	8.76	0.00	−10.56	5.17E-05	1.16E-03

Among the top coding genes that increased in expression during differentiation into NPCs and neurons were a number of members of the *HOX* gene family (discussed below), and the neuronal TF-encoding genes *POU3F3, MYT1L*, and *DLX1* (Table 2, Table 3). *MYT1l,* along with *POU3F2,* and *ASCL1*, can reprogram fibroblasts directly to neurons [31]. *POU3F2* and *ASCL1* also increased in neurons, but just failed to meet the FDR criterion for genome-wide significance.

**Table 2:** Largest fold change (FC) increase in gene expression during transition from iPSCs to NPCs.

gene	FPKM TIPS4iPSCs	FPKM TIPS4NPCs	FPKM TIPS4-C5iPSCs	FPKM TIPS4-C5NPCs	log2 FC	p-value	FDR
HOXA2	0	10.76	0	37.47	12.24	8.37E-05	1.73E-03
RP11-649A16.1	0	20.33	0	2.90	11.18	9.35E-04	1.33E-02
DCN	0.12	249.16	0	21.72	11.01	1.57E-03	2.05E-02
HOXA-AS2	0.00	15.01	0	2.03	10.74	5.61E-04	8.66E-03
HOXA3	0.00	10.53	0	5.14	10.61	1.33E-11	1.33E-09
NPR3	0.04	60.66	0	10.38	10.46	2.96E-03	3.42E-02
RGCC	0.00	2.37	0	8.93	10.14	4.39E-05	1.02E-03
PRRX1	0.22	244.97	0.04	39.91	10.02	2.51E-03	2.99E-02
DLX1	0.02	27.60	0.01	14.65	10.00	6.28E-12	6.59E-10
AC018730.3	0.00	2.73	0.00	7.11	9.94	6.59E-05	1.42E-03
NTRK2	0.01	8.74	0.04	41.85	9.86	1.83E-03	2.32E-02
RP11-266O8.1	0.00	3.71	0.00	5.45	9.84	1.70E-03	2.18E-02
PALMD	0.00	7.41	0.00	1.34	9.77	5.47E-04	8.45E-03
MKRN3	0.00	3.80	0.00	4.88	9.76	1.01E-08	5.77E-07
LUM	0.17	105.17	0.00	51.55	9.75	1.61E-07	7.08E-06
ABCC9	0.00	5.38	0.00	2.67	9.65	1.38E-08	7.50E-07
NR2F1	0.09	28.86	0.07	105.66	9.64	4.07E-08	2.00E-06
POU3F3	0.03	15.55	0.02	28.40	9.63	5.24E-22	2.04E-19
RP11-73C9.1	0.00	4.25	0.00	3.63	9.62	4.12E-03	4.49E-02
PDZRN4	0.00	15.07	0.01	1.02	9.57	3.97E-04	6.50E-03

**Table 3:** Largest fold change (FC) increase in gene expression during transition from iPSCs to Neurons.

Gene	FPKM TIPS4iPSCs	FPKM TIPS4neurons	FPKM TIPS4-C5iPSCs	FPKM TIPS4-C5neurons	log2 FC	p-value	FDR
LINC00473	0.00	40.37	0.00	12.46	12.37	2.95E-04	3.63E-03
SST	0.00	9.25	0.00	41.43	12.31	7.79E-03	4.53E-02
HOXA2	0.00	4.92	0.00	39.53	12.12	7.33E-05	1.16E-03
NEUROD6	0.00	27.12	0.00	6.10	11.70	1.47E-03	1.31E-02
NPR3	0.04	137.91	0.00	8.34	11.50	1.51E-03	1.35E-02
GS1-211B7.1	0.00	5.47	0.00	16.53	11.10	6.34E-03	3.89E-02
ABCA8	0.00	6.59	0.00	15.15	11.09	8.06E-06	1.75E-04
DIO3	0.00	12.12	0.00	9.16	11.06	3.38E-21	1.11E-18
IFI44	0.00	18.62	0.00	2.07	11.02	8.08E-03	4.67E-02
HOXB2	0.00	2.80	0.02	52.66	10.84	5.33E-03	3.44E-02
GRIA2	0.05	31.86	0.00	72.12	10.81	6.60E-07	2.00E-05
NTRK2	0.01	11.19	0.04	85.39	10.80	8.15E-04	8.16E-03
HOXA3	0.00	5.19	0.00	10.41	10.61	7.58E-12	6.73E-10
RP11-649A16.1	0.00	5.99	0.00	8.93	10.54	1.50E-03	1.34E-02
ZFHX4-AS1	0.00	17.75	0.03	32.91	10.47	7.66E-12	6.76E-10
HOXB7	0.00	2.97	0.00	9.88	10.33	3.34E-04	4.05E-03
STMN4	0.03	75.48	0.10	105.04	10.32	2.70E-23	1.25E-20
HOXB3	0.00	2.40	0.02	31.80	10.24	1.35E-03	1.23E-02
NR2F1	0.09	73.09	0.07	130.71	10.24	6.27E-09	3.11E-07
PDZRN4	0.00	5.58	0.01	19.20	10.19	1.77E-04	2.41E-03

Several lncRNAs were also among the top genes that increased in expression. However, expression levels were relatively low (FPKM <5), with the exception of LINC00473, a long intergenic non-coding RNA that overlaps with RP11-252P19.3 (see below).

Finally, there were 289 genes that showed a significant difference in expression during the transition from NPCs to neurons (168 increased; 121 decreased) (Table S4). Interestingly, several lncRNAs showed a significant increase in expression in neurons compared with NPCs, suggesting that they could be involved in neuronal maturation, rather than the initial differentiation of iPSCs into NPCs per se. These included RP11-466P24.7, RP11-64K12.10 and RP11-252P19. RP11-466P24.7 maps to the 3′-UTR of an isoform of SV2C (synaptic vesicle glycoprotein 2C); RP11-64K12.10 maps near *DISP2*, which is involved in hedgehog signaling, and RP11-252P19.3 is embedded within *SDIM1*, which is down regulated in Alzheimer’s brains and may affect NPC cell death [32], [33].

Genes that increased or decreased in expression during differentiation were subjected to pathway analysis. As seen in Table 4 and Table 5, enrichment for genes involved in SZ was found among those that increased in neurons, while genes involved in cell cycle regulation were decreased. A complete list of genes enriched for these and other functions can be found in Table S5 and Table S6. The findings show that iPSCs derived from dental pulp can be used for disease modeling neuropsychiatric disorders.

**Table 4:** Pathway Analysis for genes that increase in neurons.

Category	FunctionsAnnotation	p-Value	# Molecules
Cancer	adenocarcinoma	4.20E-12	427
Cancer	lung adenocarcinoma	4.54E-12	359
Respiratory Disease	lung adenocarcinoma	4.54E-12	359
Hereditary Disorder	Schizophrenia	8.47E-12	85
Neurological Disease	Schizophrenia	8.47E-12	85
Psychological Disorders	Schizophrenia	8.47E-12	85
Cancer	carcinoma in lung	8.21E-11	372
Respiratory Disease	carcinoma in lung	8.21E-11	372
Cancer	lung tumor	5.61E-10	377
Respiratory Disease	lung tumor	5.61E-10	377
Cancer	lung cancer	5.82E-10	375
Respiratory Disease	lung cancer	5.82E-10	375

**Table 5:** Pathway Analysis for genes that decrease in neurons.

Category	Functions Annotation	p-Value	# Molecules
Cancer	uterine serous papillary cancer	1.78E-16	59
Reproductive System Disease	uterine serous papillary cancer	1.78E-16	59
Cell Cycle	G2 phase	2.97E-11	40
DNA Replication, Recombination, Repair	metabolism of DNA	4.45E-11	49
Cell Cycle	cell cycle progression	1.25E-10	100
DNA Replication, Recombination, Repair	DNA replication	1.39E-10	34
Cell Cycle	G2/M phase	1.53E-10	33
Cell Cycle	segregation of chromosomes	2.35E-10	22
DNA Replication, Recombination, Repair	segregation of chromosomes	2.35E-10	22
Cellular Assembly and Organization	segregation of chromosomes	2.35E-10	22
Cancer	endometrial cancer	2.50E-10	65
Reproductive System Disease	endometrial cancer	2.50E-10	65
Cell Cycle	mitosis of tumor cell lines	3.59E-10	26
Cancer	colon tumor	5.19E-10	102
Gastrointestinal Disease	colon tumor	5.19E-10	102
DNA Replication, Recombination, Repair	alignment of chromosomes	5.70E-10	12
Cellular Assembly and Organization	alignment of chromosomes	5.70E-10	12
Cancer	mammary tumor	5.88E-10	144
Cell Cycle	arrest in mitosis	7.76E-10	16

### Comparison of T-iPSC and F-iPSC Neurons

RNA-seq profiles were obtained for neurons derived from the two T-iPSC lines and two F-iPSC lines. A total of 329 genes were differentially expressed at a nominally significant p-value (p<0.05), of which 63 remained significant after correcting for multiple testing (FDR <0.05; 54 expressed at a lower level in the T-iPSCs; 9 expressed at higher levels, Table S7). qPCR was used to validate the RNA-seq findings for 8 differentially expressed genes in one tooth vs fibroblast set of neurons (*ASCL1, EMX1, EMX2, FOXG1, LHX2, OTX2, TBR1, MYT1L,* and *FOXP2*). The fold change differences were consistent with the RNA-seq findings (Table S8).

No significant differences were detected for any neurotransmitter receptor or transporter gene, with the exceptions of a significant decrease in the T-iPSC neurons in the level of *GRID2* mRNA (glutamate receptor delta-2; a member of the ionotropic glutamate receptor family, and lower levels of *SLC6A1* (vesicular GABA transporter), *SLC5A7* (choline transporter) and *SLC6A5* (glycine transporter) (Table S7).

The most differentially expressed genes were *SLITRK2* and *SCUBE2. SLITRK2* codes for an integral membrane protein that shares homology with neurotrophin receptors; it has been implicated in a small subset of patients with BD and ASD [34], [35]. *SCUBE2* is expressed in the hindbrain and forms a complex with Sonic hedgehog and its receptor PTC1 to activate SHH-signaling [36], [37].

**Figure 2:**
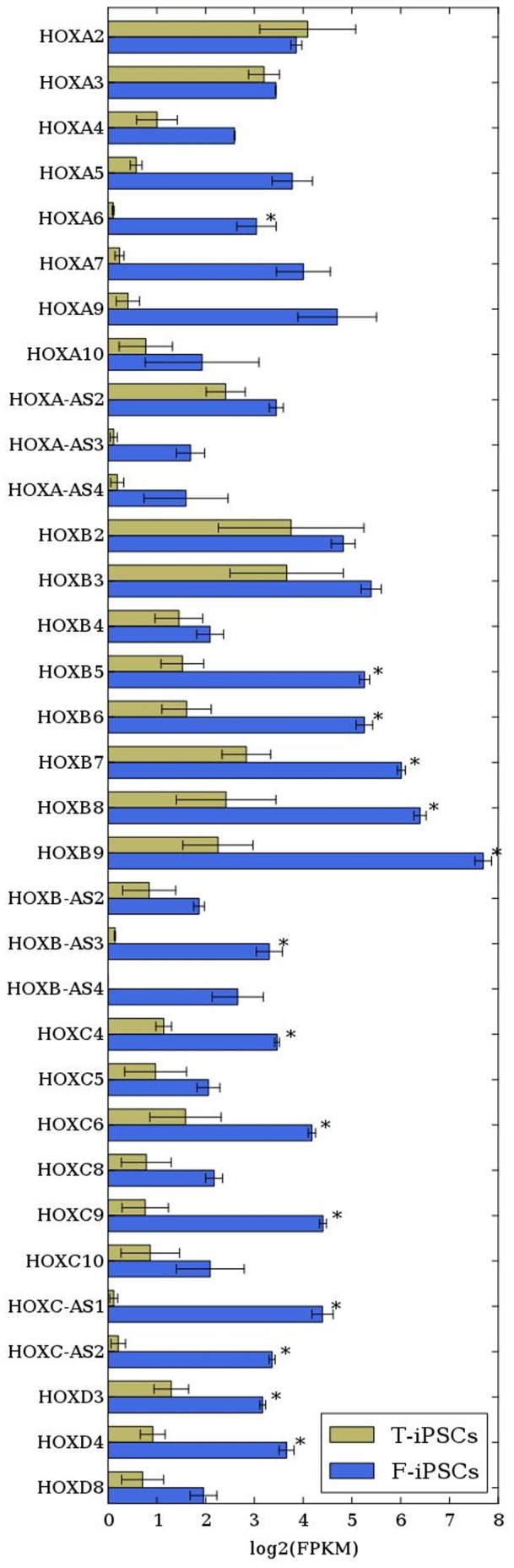
Differential expression between T-iPSCs and F-iPSCs (mean log2 fold change) for all *HOX* genes expressed at FPKM >1. Asterisk denotes FDR<0.05.

The most striking difference between the tooth and skin-derived neurons is the substantially lower expression of a number of *HOX* genes (Table S7; Figure 2). As seen in the figure, which shows the relative expression of all *HOX* genes with FPKM >1, there were 14 that showed significantly lower levels in the tooth-derived neurons (FDR<0.05; depicted by an asterisk). Seven other *HOX* genes (*HOXB-AS4, HOXA-AS3, HOXA5, HOXC8, HOXA9, HOXA7, and HOXA4*) showed decreases that were nominally significant (p<0.05; FDR>0.05). *HOX* gene expression is involved in brain patterning and is regulated by retinoic acid (RA), among other signaling pathways. Interestingly, several others genes regulated by RA are expressed at significantly lower levels in the tooth samples, including *RBP4 and RORB,* and the homeobox genes *PHOX2A*, *IRX1,* and *IRX2* (Table S7).

The nine genes that were expressed at significantly higher levels in the tooth-derived neurons (FDR<0.05) included the TFs *FOXP2, OTX1, and LHX2, as well as CNTN4, SAMD5, RP6-24A23.7, EPHA7, DNAJC25-GNG10,* and *LYSMD3.*

To examine differentially expressed genes more systematically, we subjected the data to Ingenuity Pathway Analysis (IPA). Interestingly, neurological disease/schizophrenia was the top category for genes that were expressed at higher levels in the neurons derived from teeth (nominally significant p-value of <0.05) (Table 6).

**Table 6:** Pathway analysis for differentially expressed genes (uncorrected) T-iPSC neurons vs F-iPSC neurons.

Category	Functions Annotation	p-Value	Molecules (increased in T-IPSC neurons)
Neurological Disease	Schizophrenia	3.24E-04	ATP1A2, CELF2, CHL1, CLDN5, CNTNAP2, DAB1, FOXP2, GRM3, NELL2, NTF3, SCD5, TAC1, TTR
Neurological Disease	malformation of brain	3.94E-04	ARX, CNTNAP2, EOMES, MEF2C, VLDLR
Neurological Disease	type 1 lissencephaly	9.70E-04	ARX, VLDLR
Neurological Disease	seizure disorder	2.04E-03	ARX, ATP1A2, CNTNAP2, GPR98, KCNQ3, MEF2C, PRICKLE1, TAC1
Neurological Disease	speech and language disorders	3.45E-03	ARX, FOXP1, FOXP2
Neurological Disease	epilepsy	4.12E-03	ARX, ATP1A2, CNTNAP2, KCNQ3, MEF2C, PRICKLE1, TAC1
**Category**	**Functions Annotation**	**p-Value**	**Molecules (decreased in T-iPSC neurons)**
Cellular Development	differentiation of tumor cell lines	1.29E-06	ASCL1, BTG2, CCND1, DLK1, HOXA5, JAG1, KLF4, NOTCH1, NTRK2, PRKCD, PRKD3, WNT7A, ZBTB16
Cellular Growth andProliferation	proliferation of epithelial cells	1.14E-05	CCND1, ITGB8, NOTCH1, PAX2, PRKCD, PTPRZ1, RGS4, VIP, WNT7A
Cellular Development	differentiation of cells	1.40E-05	ASCL1, BTG2, CCND1, DLK1, DLL1, FRZB, HOXA5, HOXA7, HOXA9, HS6ST1, JAG1, KLF4, MAL, NOTCH1, NTRK2, PRKCD, PRKD3, PTCH1, RGS4, TESC, WNT7A, ZBTB16
Psychological Disorders	Anxiety Disorders	1.84E-05	ADRA1A, CA14, CACNA2D3, GABRR1, GAD2, OPRM1, SLC32A1, SLC6A1
Neurological Disease	seizures	2.44E-05	ANKRD6, CA14, CACNA2D3, CYYR1, GABRR1, GAD1, GAD2, KLF10, LAMP5, PCSK1, SLC32A1, SLC6A1

## Discussion

Disease modeling using iPSCs must be carried out using readily accessible sources of somatic cells from patients for reprogramming, such as skin fibroblasts, hair keratinocytes, CD34+ leukocytes, epithelial cells found in urine, and dental pulp [11], [38]–[41]. Of these, a skin biopsy is the most invasive, which would make it the least suitable for children with developmental disorders. Obtaining hair follicles and blood for iPSC reprograming are rather non-invasive, certainly, but are not totally free of causing some degree of distress in autistic and developmentally disabled children. Deciduous teeth that are naturally shed during childhood, on the other hand, provide a source of cells for reprogramming that is not invasive, posing no additional stress to the child. Dental pulp, however, would appear to be the least convenient for researchers, since it relies on waiting for deciduous teeth to be shed. Yet, considering the time required to generate and characterize iPSC lines and the fact that children lose 20 deciduous teeth between the ages of ∼5–12, with minimal planning, collecting a library of dental pulp cells for iPSC reprogramming should not be a limiting factor.

From a biological perspective, dental pulp could prove to be a better source of iPSCs for disease modeling neuropsychiatric disorders because of its developmental origins. During early vertebrate development, embryonic ectoderm differentiates into neural and neural plate borders, and epidermal regions [42], [43]. Dental pulp contains ectomesenchyme, which is derived from ectoderm, specifically neural crest cells, while fibroblasts are derived from ectoderm that is programmed to become epidermis [42], [43]. Considering the fact that gene expression could be affected by the retention of some epigenetic marks following reprogramming that are dependent on the somatic cell of origin, an assessment of gene expression profiles using neurons derived from different reprogrammed cells could show differences that might be relevant to *in vitro* disease modeling, a question we have addressed in this paper. Expression profiling showed that neurons derived from T-iPSCs and F-iPSCs differed for some key genes, notably multiple members of the *HOX* gene families. *HOX* gene expression is involved in anterior/posterior patterning and the development of hindbrain structures. The homeobox genes *IRX1* and *IRX2,* which are also involved in brain patterning, were expressed at significantly lower levels in the neurons from T-iPSCs as well. Since both the *HOX* and *IRX* gene families are induced by RA, lower levels of expression could reflect a lower sensitivity to the RA present in the medium used during the development of NPCs.

While lower levels of expression for genes involved in hindbrain development were seen in the neurons derived from teeth, several TFs involved in the forebrain development were significantly increased as well; most notably *FOXP2, OTX1, and LHX2. FOXP2* codes for a TF involved in the development of communication and language neural networks that has been implicated in ASD [44], [45]. Considering the fact that SZ and ASD are associated with cognitive abnormalities, the decrease in expression of genes involved in hindbrain development and an increase in expression of some key forebrain TFs suggests that neurons derived from T-iPSCs may have some advantages over those derived from fibroblasts in conditions like SZ and ASD that are associated with cognitive and language impairment. In fact, pathway analysis of differentially expressed genes showing enrichment for genes involved in SZ supports this notion. On the other hand, there may be a disadvantage for disorders affecting hindbrain structures. Whether the differences in gene expression persist using other neuronal differentiation methods remains to be seen.

## Supporting Information

Figure S1 **A. Immunocytochemistry for pluripotency markers (Tra-1-60, Tra-1-80, SSEA3, SSEA4) and DAPI nuclear stain (blue) for clone TIPS4.** B. Expression of germ layer markers; AFP (endoderm), desmin (mesoderm) and β-III-tubulin (ectoderm).
(TIF)

Figure S2 **Immunocytochemistry for pluripotency markers clone TIPS4-C5.** A. Immunocytochemistry for pluripotency markers (Tra-1-60, Tra-1-80, SSEA3, SSEA4) and DAPI nuclear stain (blue) for clone TIPS4-C5.
(TIF)

Table S1 **RNA-seq statistics.** RNA-seq statistics for all T-IPSC and F-IPSC samples, and correlation coefficients for the T-IPSC samples (iPSCs, NPCs and neurons), and F-iPSCs.
(XLSX)

Table S2 **Differentially expressed genes during transition from iPSCs to NPCs.** Genes that significantly changed in expression during transition from iPSCs to NPCs for TIPS4 and TIPS4-C5 in descending order of significance.
(XLSX)

Table S3 **Differentially expressed genes during transition from iPSCs to neurons.** Genes that significantly changed in expression during transition from iPSCs to neurons for TIPS4 and TIPS4-C5 in descending order of significance.
(XLSX)

Table S4 **Differentially expressed genes during transition from NPCs to neurons.** Genes that significantly changed in expression during transition from NPCs to neurons for TIPS4 and TIPS4-C5 in descending order of log-fold change.
(XLSX)

Table S5 **Ingenuity Pathway Analysis (i).** Ingenuity Pathway Analysis for genes that increased during transition from iPSCs to neurons.
(XLS)

Table S6 **Ingenuity Pathway Analysis (ii).** Ingenuity Pathway Analysis for genes that decreased during transition from iPSCs to neurons.
(XLS)

Table S7 **Differentially expressed genes: T-iPSCs vs F-iPSCs.** Differentially expressed genes in neurons derived from T-iPSCs and F-iPSCs in descending order of significance.
(XLS)

Table S8 **Validation by quantitative real time PCR.** qPCR validation for 8 genes showing fold changes for qPCR and RNA-seq for TIPS4. qPCR carried out 3–5 times, each time point in triplicate.
(XLSX)

Text S1 **Details of methods.**
(DOCX)
