# Supplementary figures and images for: Transcriptome Comparison of Human Neurons Generated Using Induced Pluripotent Stem Cells Derived from Dental Pulp and Skin Fibroblasts
Source: PLoS One. 2013 Oct 3;8(10):e75682. doi: 10.1371/journal.pone.0075682 (PMC3789755; doi:10.1371/journal.pone.0075682)

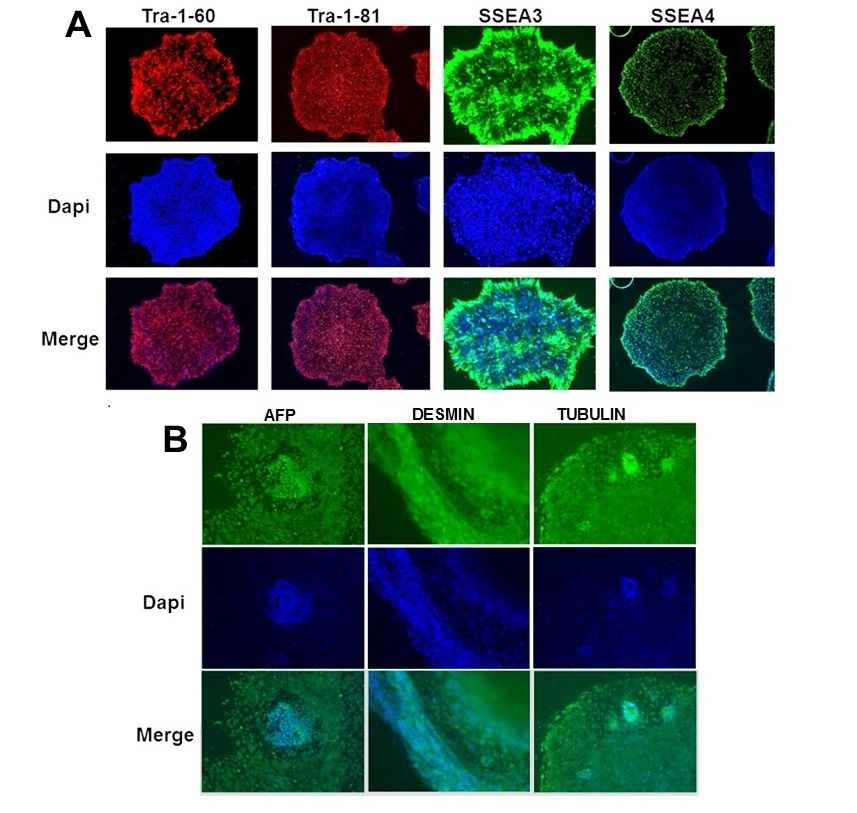

Supplement: Figure S1 — A. Immunocytochemistry for pluripotency markers (Tra-1-60, Tra-1-80, SSEA3, SSEA4) and DAPI nuclear stain (blue) for clone TIPS4. B. Expression of germ layer markers; AFP (endoderm), desmin (mesoderm) and β-III-tubulin (ectoderm). (TIF) [file pone.0075682.s001.tif]

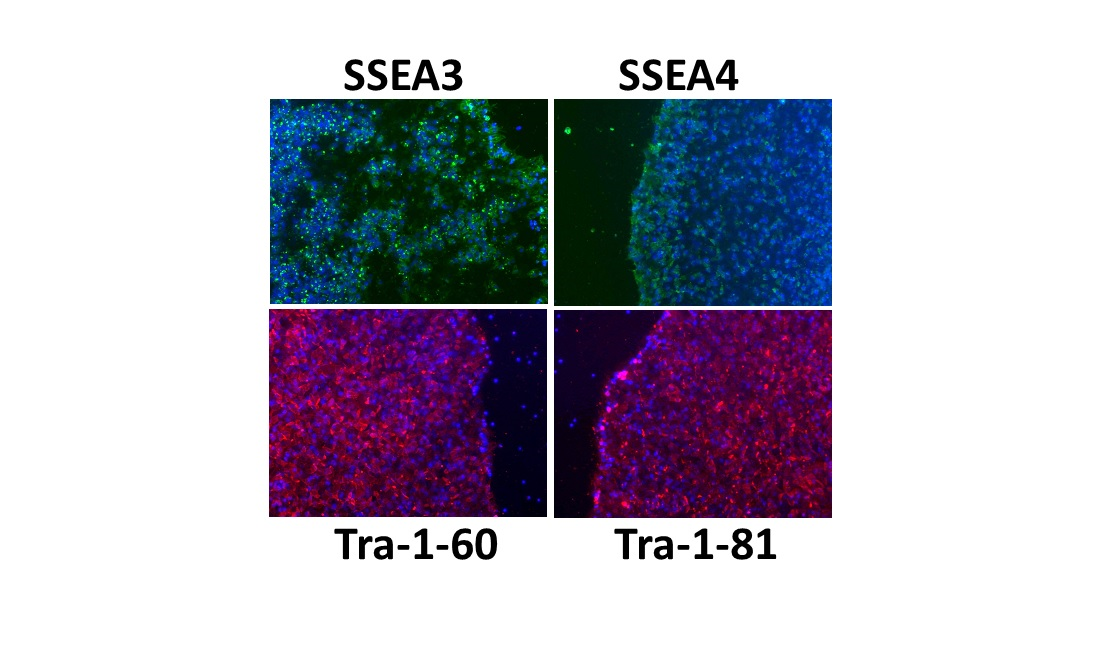

Supplement: Figure S2 — Immunocytochemistry for pluripotency markers clone TIPS4-C5. A. Immunocytochemistry for pluripotency markers (Tra-1-60, Tra-1-80, SSEA3, SSEA4) and DAPI nuclear stain (blue) for clone TIPS4-C5. (TIF) [file pone.0075682.s002.tif]
