# Supplementary material for: Transcriptome Comparison of Human Neurons Generated Using Induced Pluripotent Stem Cells Derived from Dental Pulp and Skin Fibroblasts
Source: PLoS One. 2013 Oct 3;8(10):e75682. doi: 10.1371/journal.pone.0075682 (PMC3789755; doi:10.1371/journal.pone.0075682)
Supplement: Text S1 — Details of methods. (DOCX) [file pone.0075682.s011.docx]

**Supplemental Methods**

**Harvesting Dental pulp cells and Fibroblasts**

The tooth was immediately placed in a 15 ml tube “Tooth medium” consisting of α-MEM, 10%FBS, Piperacillin (10µg/ml final concentration, Sigma cat#P8396-1G) and Ciprofloxacin (10µg/ml final concentration, Sigma cat#17850-5G-F). The sample was kept at 4^o^C until processing. Tooth germ progenitor cells (TGPCs) were processed according to (Differentiation (2008) 76:495-505) (Figure 1A). Briefly, using sterile forceps and surgical blade the mesenchyme was excised and carefully minced. Tissue was placed in a 50ml centrifuge tube 7.5mls of DMEM/F12 containing 4mg/ml Collagenase type IV and 1mM CaCl_2_. The sample was mixed at 37^o^C for 30 minutes then centrifuged at 400g for 10 minutes at 4^o^C. Excess supernatant was removed and the pellet was carefully resuspended in 2 mls of Tooth medium. The sample was transferred to a single well of a 6-well plate and placed in a humidified incubator at 37^o^C and 5% CO_2_. The sample was examined 5 days later for attachment and growth. Floating debris was removed by replacing with fresh media. Thereafter, the cells were fed fresh medium every 2 days by removing half the medium and replacing it with fresh medium. Samples were expanded in T75 flasks and frozen in liquid N_2_ in 10^6 cell aliquots (freezing medium is DMEM/F12 containing 40% FBS and 10% DMSO).

The fibroblast-derived iPSC lines used in this were described in previous publications [1-3]. Skin biopsies were performed in consenting individuals by a board-certified dermatologist. Skin biopsy samples were transferred to a small Petri dish containing 2-3 ml Skin Fibroblast Media (SFM) consisting of RPMI 1640, 10% FBS, 1% pen/strep, 10ng/ml FGF2. The sample was incubated at room temperature for 15 minutes. Medium was carefully aspirated and replaced with 1-2 ml of collagenase type II solution (3mg/ml collagenase II dissolved in DMEM High Glucose [Worthington Biochemical Corp. Lakewood, NJ: GIBCO/Invitrogen, Carlsbad, CA]). The tissue was chopped into small pieces using 2 sterile scalpels, after which they were allowed to incubate at 37^o^C for 1­2 hours depending on size. The sample was then collected in a 15ml falcon tube and washed with SFM (serum free medium). Tissue was collected by centrifugation at 1200 rpm for 4 minutes. The pelleted sample was then suspended in SFM and plated in a T12.5 ml flask at 37^o^C in 5% CO_2_ for 3 days without changing medium or manipulation, to allow fibroblasts to adhere. Then cells were subsequently fed every 2 days with RPMI 1640 containing 10% FBS until a confluent culture was obtained (~3 weeks). The cells were reprogrammed into iPSCs as described in the main methods section of the paper.

**Maintaining Human iPSCs**

iPSCs were maintained on Matrigel plates in mTeSR1 medium (Stem Cell Technologies) with daily feeding in 37^o^C/5% CO2/85% humidity.

**Neuronal differentiation**

Neurons were derived from neural progenitor cells (NPCs) as described by Marchetto et al. with slight modifications [4] iPSCs were maintained in culture until log phase growth was achieved (generally 5-6 days after passage). On day 1, the medium was changed to N2 (DMEM/F12, 1X N2; Invitrogen). The following day, Dorsomorphin was added (1μM; CALBIOCHEM). The next day, embryoid bodies (EBs) were generated. Briefly, iPSC colonies were checked again for spontaneous differentiation; patches of differentiated cells were manually removed. The fresh N2 medium with Dorsomorphin was added. Colonies were cut with by moving a 5ml glass serological pipet across the plate. Then a cell scraper was used to detach remaining cells. Cells were aliquoted to a 6 well ultra-low attachment plate (Corning), which results in a suspension of aggregates of early differentiated cells - EBs. On day 4, EBs were collected in a 15ml tube and allowed to settle via gravity for 5 minutes. Supernatant was removed and the EBs were gently resuspended in fresh N2 medium supplemented with Dorsomorphin, after which they were transferred to a new ultra-low attachment plate. From this point, EBs were fed every 2 days for 10 days. EBs were collected in a 15ml tube and allowed to settle by gravity for 5 minutes. Supernatant was removed and EBs were gently resuspended gently in NBF medium (DMEM/F12, 0.5X N2, 0.5X B27, 1% penicillin/streptomycin) plus fresh 20ng/ml FGF2 (R&D Systems). Carefully with a 10ml pipet, EBs were aliquoted to a matrigel (BD Biosciences) plate. Two days later, plates were checked for rosette formation and fed with NBF medium supplemented with fresh FGF2. Cells were fed every other day for two more feedings. Then, rosettes were carefully excised with a 26g needle and pooled in a 1.5ml tube. Accutase (ICT) was added to the rosettes for 3 minutes at 37^o^C. After incubation, rosettes were dissociated with a 1ml pipet tip, centrifuged for 2 minutes at 1,000rpm, and washed once with 1X PBS (Invitrogen). The pellet was resuspended as a single cell suspension in NBF media with fresh FGF2 (20ng/ml) and aliquoted to Poly-L-Ornithine (Sigma)/Laminin (Roche) plates, after which they differentiate into NPCs. The cells were fed every other day.

After reaching ~50% confluence, neural differentiation was induced by withdrawing FGF2. NBF media was supplemented with fresh growth factors as follows: WNT3A (100ng/ml) (R&D Systems), BDNF (10ng/ml), GDNF (10ng/ml), IGF-1 (10ng/ml) (PeproTech), and cAMP 1μM (Sigma). Cells were fed every other day.

The protocol produces a heterogenous mix of glutamatergic neurons and GABAergic neurons (~50-50 mix). Neurons were harvested after 14 days for RNA-Seq, and at day 56 for electrophysiology and staining for synaptic markers.

**Reverse transcribed PCR (RT-PCR) and quantitative real-time PCR (qPCR)**

Total RNA was extracted using a miRNeasy Kit according to the manufacturer’s instructions (Qiagen). An additional treatment with DNase1 (Qiagen, Valencia, CA) was included to remove genomic DNA. Reverse transcribed PCR (RT-PCR) was performed using a OneStep RT-PCR Kit (Qiagen, Valencia, CA) according to the manufacturer’s instructions. The cDNA was used as a template for quantitative PCR (qPCR), which was carried out using the ABI 7900HT Real-Time PCR System (Applied Biosystems, Foster City, CA). Each reaction consisted of cDNA, primers, and SYBR Green PCR Master Mix (Applied Biosystems, Foster City, CA) in an 8 μl volume. Melting curve analysis of target sequences showed that all primers used in this study generated amplicons that had a single peak, without primer-dimer artifacts. Primer concentrations were optimized prior to use in qPCR experiments. Standard curves were generated for each primer using fetal brain cDNA as a template to assess quality of qPCR experiment (slope of -3.3 indicates doubling at every cycle). Relative changes in gene expression were calculated using the 2^-∆∆Ct^ method with β2-microglobulin (β2M) as a reference gene. Each qPCR was carried out in triplicate, with each triplicate data point repeated 4-5 times. Significant differences in gene expression were assessed using a two-tailed student T-test.

Primers used in this study:

| **Primers** | **Forward** | **Reverse** |  |
| --- | --- | --- | --- |
| *HOXB9* | TTCCAGCTTGCCTGTTTCTT | TGGTCTGCAACTGGAGTGTC | |
| *FOXP2* | AGCCAGTGATTGCAGAGGAT | CTTCACGCTGAGGTTTCACA | |
| *OTX2* | CAACAGCAGAATGGAGGTCA | AGCTGGGCTCCAGATAGACA | |
| *TTR* | GTGGTATTCACAGCCAACGA | ATGAAATCCCATCCCTCGTC | |
| *CCK* | TGAGGAGTTCCCAAAGACCA | CGATACCCTCAGCTGCCTAC | |
| *GAD1* | CAAAGCCCTGATGATGGAGT | TTACAGATCCTGGCCCAGTC | |
| *CHRM2* | TACGGCTATTGCAGCCTTCT | GCAACAGGCTCCTTCTTGTC | |
| *IFITM1* | CTGATTCTGGGCATCCTCAT | AGTGCAAAGGTTGCAGGCTA | |
| *SST* | AGAAACTGACGGAGTCTGGG | GAAATTCTTGCAGCCAGCTT | |
| *EMX1* | TCACAGCCTGTCGTGAGAAC | CGAGCCCATCTCAAGAGAAC | |
| *EMX2* | CACAGAAACGGACAACATGG | CTTTAGACGAGGGTCGCTTG | |
| *FOXG1* | AGAAGAACGGCAAGTACGAGA | TGTTGAGGGACAGATTGTGGC | |
| *LHX2* | AGTTCAGGCGCAACCTCTTA | GGTGGGGCTAGTCAAGTCTG | |
| *MYT1L* | GGTGGAGACCAGTTGACCAT | ACAGCGCTCAAAAGTGGTCT | |
| *SOX2* | ACCAGCTCGCAGACCTACAT | TGGAGTGGGAGGAAGAGGTA | |
| *ASCL1* | GTCTCCCGGGGATTTTGTAT | TCTCCATCTTGGCAGAGCTT | |
| *TBR1* | GGGTCTCTGAGCTTCGTCAC | GACAGGAGGCTGTTGTAGGC | |
| *B2M* | GCTCGCGCTACTCTCTCTTT | CAATGTCGGATGGATGAAAC | |
| *OCT4* | AGATATGCAAAGCAGAAACC | ATCCTCTCGTTGTGCATAGT | |
| *VGLUT2* | ATCTTTTAGGTGCAATGGAA | CACAGCAGATAGCATACCAA | |
| *ACTA* | TTCAATGTCCCAGCCATGTA | GAAGGAATAGCCACGCTCAG | |
| *AFP* | AGCTTGGTGGTGGATGAAAC | CCCTCTTCAGCAAAGCAGAC | |
| *MAP2* | CCATGGGGCTGAGATCATTA | CTTAGCGAGTGCAGCAGTGA | |
| *Βeta-actin* | TCACCACCA CGGCCGAGCG | TCTCCTTCTGCATCCTGTCG | |
| *OCT4 plasmid* | CATTCAAACTGAGGTAAGGG | TAGCGTAAAAGGAGCAACATAG | |
| *KLF4 plasmid* | CCACCTCGCCTTACACATGAAGA | TAGCGTAAAAGGAGCAACATAG | |
| *SOX2 plasmid* | TTCACATGTCCCAGCACTACCAGA | TTTGTTTGACAGGAGCGACGAT | |
| *L-MYC plasmid* | GGCTGAGAAGAGGATGGCTAC | TTTGTTTGACAGGAGCGACGAT | |
| *LIN28 plasmid* | AGCCATATGGTAGCCTCATGTCCGC | TAGCGTAAAAGGAGCAACATAG | |

Antibodies used in this study:

| **Antibody** | **Company** | **Catalog #** |
| --- | --- | --- |
| Anti-human Tra 1-60 | eBioscience | 12-8863-80 |
| Anti-human Tra 1-81 | eBioscience | 12-8883-80 |
| AF488 Anti-mouse/human SSEA-3 | eBioscience | 53-8833-71 |
| AF488 Mouse anti SSEA-4 | BD Pharmingen | 560308 |
| Anti-Tubulin, beta III isoform | Millipore | MAB1637 |
| Desmin Ab-1 | ThermoScientific | MS-376-S |
| Anti-human/mouseα-Fetoprotein | R & D | MAB1368 |
| PSD95 (mouse) | UC Davis/NIH NeuroMab Facility | 75-028 |
| Synaptophysin(rabbit) | Millipore | AB1543P |
| Anti-GAD65/67 | Sigma | G5163 |
| Ms anti- Vglut2 | Millipore | MAB5504 |
| Rabbit neuronal class III β-tubulin | Fisher | NC9168644 |
| Sheep anti-Tyrosine Hydroxylase | Pel-Freez | P60101 |

1 Lin M, Hrabovsky A, Pedrosa E et al. Allele-biased expression in differentiating human neurons: implications for neuropsychiatric disorders. PLoS ONE 10.1371/journal.pone.0044017.

2 Pedrosa E, Sandler V, Shah A et al. Development of Patient-Specific Neurons in Schizophrenia Using Induced Pluripotent Stem Cells. J NEUROGENET 10.3109/01677063.2011.597908.

3 Lin M, Pedrosa E, Shah A et al. RNA-Seq of Human Neurons Derived from iPS Cells Reveals Candidate Long Non-Coding RNAs Involved in Neurogenesis and Neuropsychiatric Disorders. PLoS ONE 10.1371/journal.pone.0023356.

4 Marchetto MC, Carromeu C, Acab A et al. A model for neural development and treatment of rett syndrome using human induced pluripotent stem cells. CELL 10.1016/j.cell.2010.10.016.
